# Preoperative 5-Factor Frailty Index and Clavien–Dindo Grade ≥ II Complications Following Open Radical Nephrectomy: A Prospective Single-Center Cohort Study

**DOI:** 10.3390/healthcare14131886

**Published:** 2026-06-28

**Authors:** Kanza Atif, Mohammad Shoaib, Hukam Rawan Khan, Aminah Saqib, Abdal Ahmad, Eshal Atif, Sadia Qazi

**Affiliations:** 1 College of Medicine, Alfaisal University, Riyadh 11533, Saudi Arabia; katif@alfaisal.edu (K.A.); eatif@alfaisal.edu (E.A.); 2 Department of Urology, School of Medicine, Dentistry and Allied Health Sciences, PAF-IAST, Haripur 22620, Pakistan; doctr.shoaib@gmail.com; 3 Department of Urology, Kuwait Teaching Hospital, Peshawar 25120, Pakistan; hukamkhan13078@gmail.com; 4 Department of Medicine, Peshawar Medical College, Riphah International University, Peshawar Campus, Peshawar 25160, Pakistan; 172asq@gmail.com (A.S.); abdalmomand@gmail.com (A.A.); 5 Department of Anatomy, College of Medicine, Alfaisal University, Riyadh 11533, Saudi Arabia

**Keywords:** 5-Factor Frailty Index, open radical nephrectomy, renal cell carcinoma, postoperative complications, Clavien–Dindo classification, preoperative risk assessment, uro-oncology, Pakistan

## Abstract

**Highlights:**

**What are the main findings?**

- In a single-center prospective cohort of 30 patients undergoing open radical nephrectomy in Khyber Pakhtunkhwa, Pakistan, a higher preoperative 5-Factor Frailty Index (5-IFi) score was associated with increased odds of Clavien–Dindo grade ≥ II complications (odds ratio 2.1–2.35 per 1-point increase). The association persisted after adjustment for age and creatinine, although the confidence intervals approached unity.
- All observed complications were Clavien–Dindo grade II; the 5-IFi showed moderate but imprecise discrimination (AUC 0.72, 95% CI 0.53–0.88) and, at a ≥ 2 threshold, was specific (87.5%) but insensitive (50.0%).

**What are the implications of the main findings?**

- These preliminary, single-center findings suggest that the 5-IFi, which is derived from routinely available clinical records, may help flag higher-risk patients before open radical nephrectomy; however, it requires prospective validation in larger, multicenter South Asian cohorts before any clinical application.
- Given the small sample size, imprecise estimates, and exclusively grade II events, the index should be regarded as hypothesis-generating rather than an established predictor in this setting.

**Abstract:**

**Background/Objective:** Preoperative frailty assessment before open radical nephrectomy for renal cell carcinoma (RCC) is underused, and prospective data on the 5-Factor Frailty Index (5-IFi) are limited. We examined the association between the preoperative 5-IFi score and postoperative complications at a private tertiary center in Khyber Pakhtunkhwa, Pakistan. **Methods:** In this prospective cohort study, 30 adults with suspected or confirmed RCC scheduled for elective open radical nephrectomy were enrolled after ethics approval. The 5-IFi was scored preoperatively from records and medication lists. The primary outcome was any Clavien–Dindo grade ≥ II complication during the index hospitalization; secondary outcomes were length of stay and 30-day unplanned readmission. Groups were compared using Mann–Whitney U and Fisher’s exact tests. Associations were estimated by Firth penalized logistic regression with profile-likelihood confidence intervals (CIs) and receiver operating characteristic (ROC) analysis with bootstrapped CIs; adjusted models were exploratory given the sample size. **Results:** Fourteen patients (46.7%) developed a grade ≥ II complication, all grade II; nine (30.0%) were frail (5-IFi ≥ 2). The 5-IFi score was the only baseline variable significantly associated with the outcome (median 1.5 vs. 1.0; *p* = 0.030). Each 1-point increase was associated with higher odds (unadjusted OR 2.35, 95% CI 1.16–6.80; adjusted for age and creatinine, OR 2.10, 95% CI 1.00–5.91). Discrimination was moderate but imprecise (AUC 0.72, 95% CI 0.53–0.88). At the ≥2 threshold, frail patients had a higher complication rate than non-frail/pre-frail patients (77.8% vs. 33.3%; Fisher’s exact *p* = 0.046; exact OR 6.5, 95% CI 0.92–80.65), with sensitivity 50.0% and specificity 87.5%; length of stay was marginally longer in frail patients (*p* = 0.035). No grade ≥ III complications or deaths occurred. **Conclusions:** In this small single-center cohort, a higher 5-IFi score was associated with grade ≥ II complications, consistent after limited adjustment. Given the small sample, imprecise estimates, and exclusively grade II events, these findings are preliminary and hypothesis-generating. Multicenter validation is required before the 5-IFi can guide preoperative risk stratification or prehabilitation triage.

## 1. Introduction

Renal cell carcinoma (RCC) accounts for approximately 90% of kidney malignancies, with an estimated 400,000 new diagnoses and 175,000 deaths worldwide each year, and its incidence continues to rise [1,2,3]. For localized and locally advanced disease, radical nephrectomy remains the accepted curative standard according to current AUA guidelines [4]. As the surgical population ages, urologic oncologists increasingly operate on patients with substantial comorbidity, yet the preoperative tools routinely used to assess fitness for major extirpative surgery have not kept pace with this shift.

Preoperative risk assessment has long relied on chronological age and the American Society of Anesthesiologists (ASA) physical status classification. Both are broad signals, but the ASA score has well-documented inter-rater reliability limitations and does not capture physiological reserve or tolerance to surgical stress [5]. Neither tool measures frailty—a syndrome of diminished physiological reserve and heightened vulnerability to stressors, first formally characterized as a phenotype by Fried et al. (2001) [6]. Preoperative frailty has since been established as a predictor of postoperative complications, prolonged stay, and mortality across surgical disciplines, including oncologic surgery [7,8,9].

Full geriatric frailty assessment is impractical in most preoperative settings. The 5-Factor Frailty Index (5-IFi), derived by Subramaniam et al. from American College of Surgeons NSQIP data, reduces frailty assessment to five variables extractable from any standard clinical record: pharmacologically managed hypertension, diabetes mellitus, and chronic obstructive pulmonary disease; a history of congestive heart failure; and dependence on activities of daily living [10]. It correlates strongly with the original eleven-variable index and has been examined against other risk-stratification tools in urologic oncology cohorts [10,11]. However, prospective data evaluating its performance in a dedicated open radical nephrectomy population are scarce. Therefore, we conducted an exploratory prospective cohort study to examine this.

This study evaluated the association between the preoperative 5-IFi score and the incidence of postoperative complications, defined as Clavien–Dindo grade ≥ II during index hospitalization, in patients undergoing open radical nephrectomy for RCC. The secondary outcomes included length of hospital stay, 30-day unplanned readmission, and 30-day mortality. Given the exploratory design and modest sample size, the findings are intended to be hypothesis-generating.

## 2. Materials and Methods

### 2.1. Study Design and Patient Selection

This prospective observational cohort study was conducted following Institutional Review Board (IRB) approval (Prime/IRB/2025-1164, dated 5 June 2025) at a single private tertiary center (Kuwait Teaching Hospital, Peshawar, Khyber Pakhtunkhwa, Pakistan) and is reported in accordance with the STROBE guidelines for observational studies [12]. Consecutively eligible adult patients (aged ≥ 18 years) with suspected or confirmed RCC who were scheduled for elective open radical nephrectomy were screened. Inclusion required written informed consent and availability for a 30-day postoperative follow-up. Patients requiring emergency surgery, presenting with clinical metastatic disease (cM1), or undergoing nephron-sparing surgery were excluded, as were patients with severe neurological deficits that precluded accurate baseline functional assessment.

### 2.2. Frailty Assessment (5-IFi)

The preoperative physiological reserve was assessed using the 5-IFi [10]. Trained clinical data abstractors reviewed each patient’s medical records, active problem lists, and current medication regimens prior to surgery. One point was assigned for the documented presence of each of the following: hypertension requiring antihypertensive medication, diabetes mellitus (type 1 or 2) requiring oral hypoglycemics or insulin, chronic obstructive pulmonary disease or chronic bronchitis requiring respiratory medication, a history of congestive heart failure, and dependent functional status. Functional status was assessed using a standardized preoperative questionnaire based on the Katz Index of Activities of Daily Living [13]. Patients requiring assistance with one or more of the six ADLs were assigned points. Cumulative scores (range 0–5) stratified the cohort into non-frail (0), pre-frail (1), and frail (≥2) categories [10].

### 2.3. Data Collection and Outcome Measures

Baseline demographic variables (age, sex, BMI, smoking status), laboratory values (creatinine, hemoglobin, albumin), and operative data were collected. The primary outcome was any Clavien–Dindo grade ≥ II complication during the index hospitalization, graded according to the Dindo–Demartines–Clavien classification [14,15]. Secondary outcomes included total length of hospital stay, 30-day unplanned readmission, and 30-day mortality. Post-discharge events were captured through standardized research coordinator contact at 7, 14, and 30 days postoperatively; these were self-reported and were not graded using the Clavien–Dindo system.

### 2.4. Statistical Analysis

Continuous variables are reported as medians with interquartile ranges (IQR) and categorical variables as frequencies and percentages. Between-group comparisons were performed using the Mann–Whitney U test for continuous variables and Fisher’s exact test for categorical variables. The 5-IFi was analyzed as both a continuous score and at a prespecified frailty threshold of ≥2. To estimate the association between the continuous 5-IFi score and the primary outcome, Firth penalized logistic regression was used to reduce small-sample bias and separation [16]; three nested models were fitted (unadjusted, adjusted for age, and adjusted for age and creatinine. Confidence intervals for odds ratios were derived from the penalized profile likelihood; the threshold odds ratio and its confidence interval were obtained from Fisher’s exact test (conditional, exact method); and the area under the ROC curve (AUC) [17] was accompanied by a 3000-iteration bootstrap percentile confidence interval [18]. Given the cohort size (*N* = 30, 14 events; events-per-variable 4.7), the adjusted models were exploratory; no adjustment for multiple comparisons was applied, and no a priori sample size calculation was performed. The ASA class was not available and could not be included. Analyses were performed using Python (version 3.11; Python Software Foundation, Beaverton, OR, USA), using the pandas (version 2.1.0), SciPy (version 1.11.0), and scikit-learn (version 1.3.0) packages. Statistical significance was set at *p* < 0.05, with emphasis on effect estimates and their precision rather than on significance alone.

## 3. Results

### 3.1. Baseline Demographics and Clinical Characteristics

Thirty patients were included in the analytical cohort of this study. The median age was 52.0 years (IQR 45.0–58.0), median BMI was 22.2 kg/m^2^ (IQR 19.9–25.4), and median preoperative 5-IFi score was 1.0 (IQR 1.0–2.0). Nine patients (30.0%) were classified as frail (5-IFi ≥ 2), and 21 (70.0%) were classified as non-frail or pre-frail (5-IFi < 2). The frequency distribution of the five 5-IFi components is provided in Supplementary Table S1. Hypertension was the most prevalent comorbidity (40.0%), followed by functional dependence (36.7%) and diabetes mellitus (33.3%), whereas COPD and congestive heart failure were the least common (10.0% each). Baseline characteristics stratified by frailty status are shown in Supplementary Table S2. Hypertension (88.9% vs. 19.0%, *p* = 0.001) and diabetes (66.7% vs. 19.0%, *p* = 0.030) were more frequent among frail patients, whereas age, BMI, sex, and creatinine did not differ significantly. The baseline characteristics stratified by the primary outcome are presented in Table 1. The 5-IFi score was the only baseline variable that differed significantly between patients with and without a complication (median 1.5 vs. 1.0; *p* = 0.030); age (*p* = 0.096), BMI (*p* = 0.803), creatinine (*p* = 0.754), hypertension (*p* = 0.457), and diabetes (*p* = 1.000) did not, nor did sex, smoking status, hemoglobin, albumin, or tumor size and clinical stage (all *p* > 0.05; Table 1).

### 3.2. Postoperative Morbidity and Secondary Outcomes

**Figure 1:**
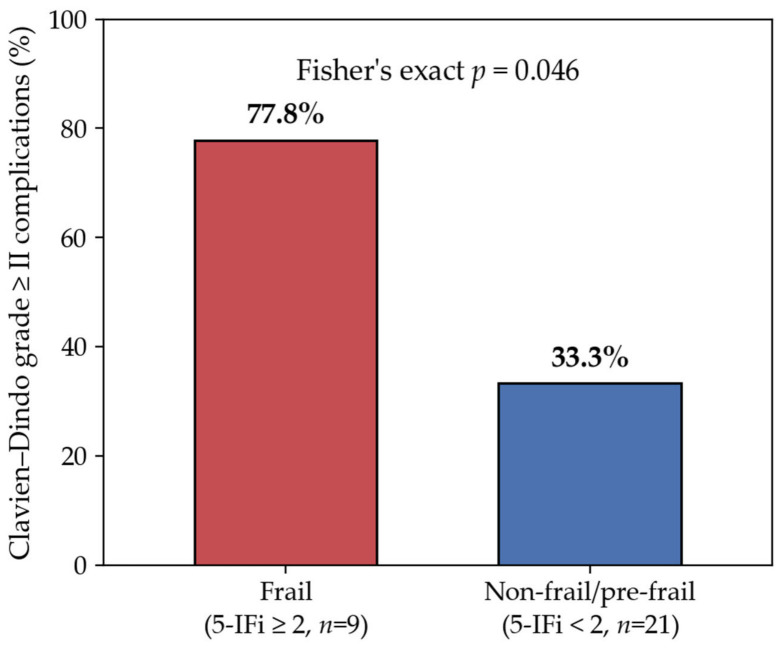
Grade ≥ II complication rate by preoperative frailty status. Frail patients (5-IFi ≥ 2, *n* = 9) experienced complications at 77.8% versus 33.3% in non-frail/pre-frail patients (5-IFi < 2, *n* = 21); Fisher’s exact *p* = 0.046.

Clavien–Dindo grade ≥ II complications occurred in 14 patients (46.7%). All 14 were grade II, reflecting pharmacological management (including antibiotics and blood transfusion); there were no complications of grade ≥ III, no reoperations, and no in-hospital or 30-day deaths (Supplementary Table S3). Three patients (10.0%) required ICU admission. When stratified by frailty (Table 2), frail patients experienced complications more often than non-frail/pre-frail patients (77.8% vs. 33.3%; Fisher’s exact *p* = 0.046) and had a marginally longer length of stay (median 3.0 [IQR 3.0–4.0] vs. 3.0 [2.0–3.0] days; *p* = 0.035). ICU admission (22.2% vs. 4.8%; *p* = 0.207) and 30-day unplanned readmission (22.2% vs. 0%; *p* = 0.083) were numerically higher among frail patients but did not reach statistical significance. Post-discharge complications within 30 days were reported by 21 patients (70.0%) via research-coordinator contact; these were self-reported, were not graded by the Clavien–Dindo system and were therefore not stratified by frailty (Figure 1).

### 3.3. Discrimination and Threshold Analysis

**Figure 2:**
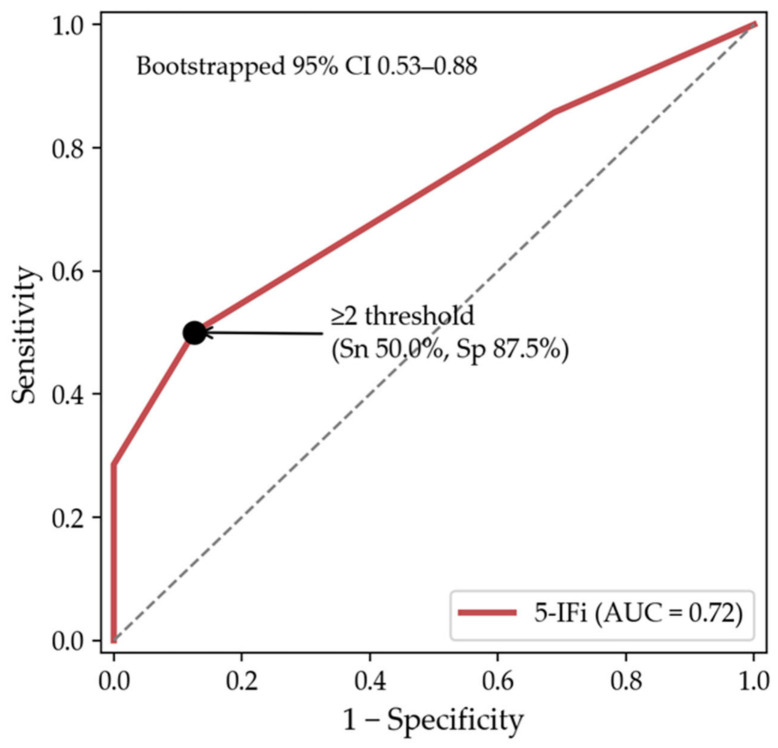
Receiver operating characteristic (ROC) curve for the preoperative 5-IFi score as a marker of complications of grade ≥ II. AUC = 0.72 (bootstrapped 95% CI 0.53–0.88). At the ≥2 threshold, the sensitivity was 50.0% and the specificity was 87.5% (marked point). The dashed line denotes the absence of discrimination.

In the Firth penalized logistic regression, each 1-point increase in the 5-IFi score was associated with higher odds of the primary outcome across all three nested models: unadjusted OR 2.35 (95% CI 1.16–6.80), age-adjusted OR 2.20 (95% CI 1.02–6.44), and age- and creatinine-adjusted OR 2.10 (95% CI 1.00–5.91) (Table 3; Supplementary Table S4). The point estimate shifted only modestly across the models, although the lower confidence bound approached unity after adjustment. Neither age (OR 1.01 per year) nor creatinine (OR 0.60 per mg/dL) was independently associated with the outcomes. At the prespecified ≥2 threshold, frailty was associated with a crude odds ratio of 7.0 (exact conditional OR 6.5; 95% CI 0.92–80.65; Fisher’s exact *p* = 0.046). The wide interval, which includes 1.0, reflects the small number of frail patients (*n* = 9) and the resulting imprecision. At this threshold, the 5-IFi had a sensitivity of 50.0% and specificity of 87.5% for the primary outcome. ROC analysis of the continuous 5-IFi score yielded an AUC of 0.72 (bootstrapped 95% CI 0.53–0.88), indicating moderate but imprecise discrimination (Figure 2).

## 4. Discussion

In this small, single-center, prospective cohort study, a higher preoperative 5-IFi score was associated with Clavien–Dindo grade ≥ II complications after open radical nephrectomy. Each 1-point increase in the score was associated with roughly a doubling of the odds of a complication (unadjusted OR 2.35; adjusted for age and creatinine OR 2.10), and the association was consistent across nested models, although the confidence intervals approached unity after adjustment. Notably, the aggregate 5-IFi score, but none of the individual baseline variables, including age, BMI, hypertension, diabetes, and creatinine, differed significantly between patients with and without complications (Table 1). This pattern is compatible with the deficit-accumulation interpretation of frailty [19], in which co-occurring deficits may carry information that individual comorbidities do not; however, the small sample precludes strong inference, and the observation is hypothesis-generating.

### 4.1. Comparison with the Uro-Oncology Literature

Our findings are directionally consistent with the larger retrospective literature, but the magnitude of the association is not directly comparable because of differences in endpoint definitions. The largest series, the NSQIP analysis by Ayoub et al. (*N* = 36,682), reported higher odds of prolonged stay, grade ≥ 4 complications, and 30-day mortality among frail patients (OR 1.85) [20]. Our per-point odds ratio (≈2.1) is of a similar order, whereas our threshold odds ratio (exact OR 6.5) is larger but highly imprecise (95% CI 0.92–80.65) and rests on only nine frail patients; it should not be over-interpreted. Importantly, our primary endpoint (grade ≥ II, and in practice entirely grade II) is broader and less severe than the grade ≥ 4 or mortality endpoints used in several NSQIP analyses; therefore, a larger apparent effect size is expected and does not indicate a stronger underlying effect. Our results are consistent with frailty–morbidity associations reported across major urologic oncology, including radical prostatectomy [21] and minimally invasive partial nephrectomy [22]. In a multi-procedure NSQIP analysis including patients undergoing radical prostatectomy, partial nephrectomy, radical nephrectomy, and radical cystectomy, the modified frailty index demonstrated only modest discrimination for major perioperative complications, with C-statistics ranging from 0.531 to 0.607 [23], in line with the moderate, imprecise discrimination observed here. Our results are consistent with the competing risk perspective of Rosiello et al. [24]. Schmeusser and Master argued that the five-component structure is more clinically practical than its eleven-variable predecessor [25]; our data provide preliminary prospective signal consistent with that view in an open-surgery population, while underscoring that the estimate is imprecise.

### 4.2. Open Surgery Context

Open radical nephrectomy carries higher baseline morbidity than minimally invasive approaches [26,27]. In a national analysis, Pascal et al. found that open surgery was independently associated with higher rates of postoperative anemia, acute renal failure, sepsis, and bleeding [27]. It is plausible that the greater physiological demand of open surgery makes the diminished reserve more consequential, which could contribute to the association observed here. However, we did not measure inflammatory, metabolic, or physiological reserve markers and, therefore, cannot test any mechanistic explanation. Because this cohort contained no minimally invasive comparator, we also cannot assess whether the frailty signal would be attenuated with less invasive surgery; these mechanistic and comparative questions are beyond the scope of the present data.

### 4.3. Cohort Characterization and Regional Context

The cohort was relatively young (median age 52 years) and lean (median BMI 22.2 kg/m^2^), and the most frequent frailty components were hypertension (40.0%) and functional dependence (36.7%), followed by diabetes (33.3%) (Table S1). Therefore, the frailty captured here was not dominated by a single deficit; cardiometabolic and functional deficits contributed in similar proportions. Although Pakistan has a high population burden of diabetes and metabolic syndrome [28,29,30], the present surgical cohort was not characterized by obesity or a uniformly high metabolic-risk profile, and we caution against extrapolating population-level metabolic epidemiology to this small surgical sample. Whether the ≥2 threshold performs differently in South Asian surgical populations cannot be determined from 30 patients and would require a dedicated and adequately powered study.

### 4.4. The Post-Discharge Complication Gap

A higher proportion of patients reported post-discharge complications (70.0%) than those who experienced in-hospital grade ≥ II complications (46.7%). However, post-discharge events were self-reported and were not graded by the Clavien–Dindo system; therefore, the two figures are not directly comparable, and the post-discharge rate is best regarded as hypothesis-generating. This pattern is consistent with prior work identifying the early post-discharge period as one of continued vulnerability after nephrectomy [31] and raises the possibility that readmission-based metrics underestimate community morbidity in resource-limited settings; rigorous, graded post-discharge data are required to establish this.

### 4.5. Clinical Implications

Because this study only evaluated an association and did not test any intervention, its clinical implications are limited and provisional. If the association is confirmed in larger cohorts, a simple, records-based screen such as the 5-IFi could, in principle, help identify patients for closer perioperative attention or structured prehabilitation [32,33] and could inform individualized preoperative discussion [34,35]. However, we did not assess prehabilitation, decision-making, consent quality, or any patient-centered outcomes, and the index was neither calibrated nor externally validated in this study. Therefore, we do not recommend routine clinical use at this stage.

### 4.6. Limitations

This study had several important limitations. First, it is a small, single-center convenience sample (*N* = 30) with 14 outcome events, yielding an event-per-variable ratio of approximately 4.7—well below the conventional minimum of 10—so the adjusted models are exploratory and the estimates are imprecise. No a priori sample size calculation was performed; the study was underpowered, and a cohort of approximately 80–100 patients would be required to estimate the observed per-point effect with adequate precision. Second, the estimates were imprecise: the threshold odds-ratio confidence interval included 1.0, and the lower bound of the AUC confidence interval (0.53) was close to chance; therefore, the discriminatory performance could not be regarded as established. Third, only age and creatinine levels were entered as covariates, given the limited number of events. Additional baseline variables (sex, smoking, hemoglobin, albumin, tumor size, and clinical stage) did not differ between groups; however, the events-per-variable constraint precluded their inclusion in the adjusted models, and nutritional status may add predictive value in larger studies [36]. Other factors, such as ASA class, tumor complexity, operative duration, intraoperative blood loss, transfusion practices, surgeon volume, sarcopenia, and cognition, were not captured, leaving residual confounding that cannot be excluded. Fourth, complication grading was based on the documented treatment: all events corresponded to grade II (pharmacological management, including antibiotics and blood transfusion), and three patients required ICU admission, but organ-specific intensive-care criteria were not separately adjudicated, and intraoperative or anticipated transfusions were not consistently distinguished from those treating a postoperative complication, both of which could misclassify the outcome. Model calibration was not assessed, as it was not meaningfully estimable in this sample size. Fifth, multiple comparisons were not adjusted for. Sixth, the data abstractors were not blinded to the outcomes, and the inter-rater reliability was not formally assessed. Seventh, post-discharge complications were self-reported, ungraded, and subject to recall bias, and the follow-up period was limited to 30 days. Eighth, the exclusion of patients with severe neurological deficits may have removed some of the frailest individuals, potentially biasing the association toward the null. Finally, the 5-IFi was derived and validated in North American populations but has not been validated locally; these results require external, preferably multicenter, validation before any clinical application.

## 5. Conclusions

In this small, single-center prospective cohort of patients undergoing open radical nephrectomy, a higher preoperative 5-IFi score was associated with Clavien–Dindo grade ≥ II complications, and this association persisted after limited adjustment for age and creatinine. Individual baseline variables did not differ by outcome, whereas the aggregate score did, which is a pattern compatible with, though not proof of, a deficit-accumulation model of frailty. All complications were grade II, and the estimates were imprecise. These findings are preliminary and hypothesis-generating; they do not establish the 5-IFi as an independent predictor or calibrated risk tool in this population. Adequately powered multicenter prospective studies with richer covariate capture, formal calibration, and graded post-discharge follow-up are needed before the 5-IFi can be recommended for routine preoperative risk stratification, prehabilitation triage, or surgical consent in open radical nephrectomy.

## Figures and Tables

**Table 1:** Baseline characteristics stratified by the primary outcome.

Characteristic	Primary Outcome (*n* = 14)	No Primary Outcome (*n* = 16)	*p*-Value
Age, years	56.5 (48.2–60.8)	49.5 (44.5–53.5)	0.096
Male sex, *n* (%)	10 (71.4%)	9 (56.2%)	0.466
BMI, kg/m^2^	22.5 (21.6–23.5)	21.7 (19.4–26.0)	0.803
Ever-smoker, *n* (%)	3 (21.4%)	4 (25.0%)	1.000
5-IFi score	1.5 (1.0–2.75)	1.0 (0.0–1.0)	0.030 *
Creatinine, mg/dL	1.15 (0.80–1.50)	1.35 (0.90–1.42)	0.754
Hemoglobin, g/dL	12.6 (12.1–12.9)	12.3 (11.3–13.4)	1.000
Albumin, g/dL	3.7 (3.3–4.2)	3.7 (3.1–3.9)	0.279
Hypertension, *n* (%)	7 (50.0%)	5 (31.2%)	0.457
Diabetes mellitus, *n* (%)	5 (35.7%)	5 (31.2%)	1.000
Tumor size, cm	8.2 (3.5–9.3)	8.0 (5.6–9.2)	1.000
Clinical stage cT2–T4, *n* (%)	7 (50.0%)	9 (56.2%)	1.000

Note: Continuous variables: median (IQR), Mann–Whitney U; categorical: *n* (%), Fisher’s exact test. * *p* < 0.05.

**Table 2:** Postoperative outcomes stratified by frailty status.

Outcome	Frail (5-IFi ≥ 2, *n* = 9)	Non-Frail (5-IFi < 2, *n* = 21)	*p*-Value
Grade ≥ II morbidity, *n* (%)	7 (77.8%)	7 (33.3%)	0.046 *
Length of stay, days	3.0 (3.0–4.0)	3.0 (2.0–3.0)	0.035 *
ICU admission, *n* (%)	2 (22.2%)	1 (4.8%)	0.207
30-day readmission, *n* (%)	2 (22.2%)	0 (0.0%)	0.083
Post-discharge complication ≤ 30 d, *n* (%)	21 (70.0%) †	—	—

* *p* < 0.05. † Self-reported across all 30 patients; not Clavien–Dindo graded; not stratified by frailty.

**Table 3:** Firth penalized regression, threshold, and ROC analyses of the 5-IFi for the primary outcome.

Model/Variable	OR	95% CI
5-IFi score (continuous, per point)		
Unadjusted	2.35	1.16–6.80
Adjusted for age	2.20	1.02–6.44
Adjusted for age + creatinine	2.10	1.00–5.91
Covariates (fully adjusted model)		
Age, per year	1.01	0.93–1.11
Creatinine, per mg/dL	0.60	0.06–5.75
Threshold (5-IFi ≥ 2 vs. <2)		
Exact (conditional) OR	6.5	0.92–80.65 ‡
ROC analysis		
AUC	0.72	0.53–0.88 §
Sensitivity at ≥2	50.0%	—
Specificity at ≥2	87.5%	—

Notes: Odds ratios for the continuous 5-IFi score are per 1-point increase. ‡ Exact conditional CI; Fisher’s exact *p* = 0.046. § Bootstrap percentile CI (3000 iterations). All other CIs are penalized profile likelihood intervals. OR: odds ratio; CI: confidence interval; AUC: area under the ROC curve.

## Data Availability

The data supporting the findings of this study are available at https://doi.org/10.6084/m9.figshare.32148319.

## Supplementary Materials

The following supporting information can be downloaded at: https://www.mdpi.com/article/10.3390/healthcare14131886/s1, Table S1: frequency distribution of 5-IFi components; Table S2: baseline characteristics by frailty status; Table S3: postoperative complications by Clavien–Dindo grade; Table S4: penalized (Firth) logistic regression with profile-likelihood confidence intervals.
