# Supplementary material for: Preoperative 5-Factor Frailty Index and Clavien–Dindo Grade ≥ II Complications Following Open Radical Nephrectomy: A Prospective Single-Center Cohort Study
Source: Healthcare (Basel). 2026 Jun 28;14(13):1886. doi: 10.3390/healthcare14131886 (PMC13361096; doi:10.3390/healthcare14131886)
Supplement: Supplementary file 1 [file healthcare-14-01886-s001.zip › healthcare-4329069-supplementary.pdf]

Supplementary Materials

Preoperative 5-Factor Frailty Index and Clavien–Dindo Grade ≥ II Complications Following Open Radical Nephrectomy: A Prospective Single-Centre Cohort Study

Section S1. Statistical methodology

S1.1. Firth penalized logistic regression. In small samples or with rare outcomes, maximum-likelihood logistic regression frequently encounters separation or monotone likelihood, producing infinite or heavily biased estimates. Firth penalized likelihood (Jeffreys-prior penalty) reduces small-sample bias and stabilizes estimation. Odds-ratio confidence intervals were derived from the penalized profile likelihood.

S1.2. Interval estimation. The threshold odds ratio and its confidence interval were obtained from Fisher's exact test (conditional, exact method). The area under the ROC curve was accompanied by a 3,000-iteration bootstrap percentile confidence interval (2.5th–97.5th percentile). Analyses used Python (pandas, SciPy, scikit-learn).

Section S2. Supplementary tables

Table S1. Frequency distribution of 5-Factor Frailty Index (5-IFi) components (*N* = 30).

5-IFi component	Description	Frequency, *n* (%)
Hypertension	Requiring antihypertensive medication	12 (40.0%)
Functional dependence	Dependent functional status (Katz ADL–based determination)	11 (36.7%)
Diabetes mellitus	Type 1 or 2; oral hypoglycaemics or insulin	10 (33.3%)
COPD / chronic bronchitis	Requiring respiratory medication	3 (10.0%)
Congestive heart failure	Documented history of CHF	3 (10.0%)

Note. Each component scored present (1) or absent (0) from medical-record, problem-list and medication review by trained abstractors. Functional status reflects the abstractor's clinical determination informed by a Katz-based ADL questionnaire. ADL, activities of daily living; COPD, chronic obstructive pulmonary disease.

Table S2. Baseline characteristics by frailty status (5-IFi ≥ 2 vs < 2).

Characteristic	Frail (5-IFi ≥ 2, *n* = 9)	Non-frail (5-IFi < 2, *n* = 21)	*p*-value
Age, years	58.0 (49.0–60.0)	50.0 (43.0–55.0)	0.118
Male sex, *n* (%)	6 (66.7%)	13 (61.9%)	1.000
BMI, kg/m²	22.1 (21.5–23.6)	22.4 (19.6–25.7)	0.892
Ever-smoker, *n* (%)	2 (22.2%)	5 (23.8%)	1.000
Creatinine, mg/dL	1.00 (0.80–1.60)	1.30 (0.90–1.40)	0.982
Haemoglobin, g/dL	12.6 (12.1–13.0)	12.3 (11.7–13.0)	0.700
Albumin, g/dL	3.7 (3.3–4.2)	3.6 (3.2–4.0)	0.634
Diabetes mellitus, *n* (%)	6 (66.7%)	4 (19.0%)	0.030*
Hypertension, *n* (%)	8 (88.9%)	4 (19.0%)	0.001*
Tumour size, cm	7.9 (3.3–11.2)	8.3 (5.6–9.3)	0.768
Clinical stage cT2–T4, *n* (%)	5 (55.6%)	11 (52.4%)	1.000
Grade ≥ II morbidity, *n* (%)	7 (77.8%)	7 (33.3%)	0.046*

*Note. Continuous variables: median (IQR), Mann–Whitney U; categorical: n (%), Fisher's exact test. ASA class was not recorded in the dataset and is therefore not reported. *p < 0.05.*

Table S3. Postoperative complications by Clavien–Dindo grade.

**Clavien–Dindo grade**	**Representative events (treatment)**	***n***
Grade II	UTI, pneumonia (antibiotics); bleeding (blood transfusion)	14
Grade III–V	—	0
Total grade ≥ II		14

Note. All 14 grade ≥ II complications were Grade II (pharmacological management, including antibiotics and blood transfusion). Three patients required ICU admission (monitoring); there were no reoperations and no in-hospital or 30-day deaths.

Table S4. Penalized (Firth) logistic regression with profile-likelihood confidence intervals.

**Predictor (per 1-point 5-IFi)**	**OR**	**95% CI**	**Model**
5-IFi, unadjusted	2.35	1.16–6.80	Unadjusted
5-IFi, adjusted for age	2.20	1.02–6.44	Model 2
5-IFi, adjusted for age + creatinine	2.10	1.00–5.91	Model 3 (final)
Age, per year (Model 3)	1.01	0.93–1.11	Model 3 (final)
Creatinine, per mg/dL (Model 3)	0.60	0.06–5.75	Model 3 (final)

Note. Firth penalized likelihood with profile-likelihood confidence intervals. Odds ratios for the 5-IFi score are per 1-point increase. CI, confidence interval.
